# Non-Intrusive Load Monitoring for Residential Appliances with Ultra-Sparse Sample and Real-Time Computation

**DOI:** 10.3390/s21165366

**Published:** 2021-08-09

**Authors:** Minzheng Hu, Shengyu Tao, Hongtao Fan, Xinran Li, Yaojie Sun, Jie Sun

**Affiliations:** 1 Department of Light Sources and Illuminating Engineering, Fudan University, Shanghai 200433, China; 19210860057@fudan.edu.cn (M.H.); 19210720062@fudan.edu.cn (S.T.); 18110720036@fudan.edu.cn (H.F.); 20110720023@fudan.edu.cn (X.L.); 20110720093@fudan.edu.cn (J.S.); 2 Shanghai Engineering Research Center for Artificial Intelligence and Integrated Energy System, Fudan University, Shanghai 200433, China; 3 Institute for Six-Sector Economy, Fudan University, Shanghai 200433, China

**Keywords:** non-intrusive load monitoring (NILM), wavelet, overshoot multiple, weighted K-nearest neighbor (KNN), sparse sample, real-time computation

## Abstract

To achieve the goal of carbon neutrality, the demand for energy saving by the residential sector has witnessed a soaring increase. As a promising paradigm to monitor and manage residential loads, the existing studies on non-intrusive load monitoring (NILM) either lack the scalability of real-world cases or pay unaffordable attention to identification accuracy. This paper proposes a high accuracy, ultra-sparse sample, and real-time computation based NILM method for residential appliances. The method includes three steps: event detection, feature extraction and load identification. A wavelet decomposition based standard deviation multiple (WDSDM) is first proposed to empower event detection of appliances with complex starting processes. The results indicate a false detection rate of only one out of sixteen samples and a time consumption of only 0.77 s. In addition, an essential feature for NILM is introduced, namely the overshoot multiple (which facilitates an average identification improvement from 82.1% to 100% for similar appliances). Moreover, the combination of modified weighted K-nearest neighbors (KNN) and overshoot multiples achieves 100% appliance identification accuracy under a sampling frequency of 6.25 kHz with only one training sample. The proposed method sheds light on highly efficient, user friendly, scalable, and real-world implementable energy management systems in the expectable future.

## 1. Introduction

Energy conservation is always on the agenda of international organizations, commonweal groups, and regional governments worldwide. In response to both soaring energy demands and their consequential environmental impacts, the goal of carbon neutrality has been proposed recently to balance the emission and absorption of greenhouse gases [1]. The building load is the largest energy consumption end, accounting for an average of over 40% electricity in developed countries [2]. In this regard, building loads such as residential, education, office, healthcare, and industrial loads are emerging as critical consumers in energy consumption sectors [3]. The energy demand of residential loads issued by the U.S Energy Information Administration in 2050 will increase by 211% compared with that of 2020 [4]. Meanwhile, residential loads are promising in energy conservation; it is projected that every resident can save $500 for two years by inducing energy-saving investments [5]. Herein, energy management systems (EMS), related to residential loads, is emerging as a vigorous field with good prospects and has been attracting widespread attention [6,7,8] due to its extensive but fundamental support in load monitoring, smart homes, energy conservation, prognostic demand response, etc. [3,9,10,11,12].

With the dual-purpose of monitoring and managing residential loads, prerequisites such as real-time load sensing, identification, and analysis are indispensable for further residential EMS. Currently, there are two possible residential EMS solutions: (a) Intrusive Load Monitoring (ILM) and (b) Non-Intrusive Load Monitoring (NILM). In ILM (i.e., a hardware-based approach), sensors are attached to each target appliance. The large number of hardware devices required for ILM makes the installation process time-consuming and cost-intensive while simultaneously contributing to an accurate load identification performance. On the other hand, NILM is a software-based approach. It requires only one sensor at the load bus terminal, and therefore the installation process is simplified and the corresponding costs are reduced [13,14]. A typical NILM software framework embraces the following three steps: event detection, feature extraction and load identification [15].

Event detection refers to the process of determining whether an appliance is turned on or off for positioning purposes. There are currently two paradigms for event detection [16]. One is to directly detect the trip point of the measured signal and calculate the difference between any two adjacent characteristic indicators (such as active power) in the collected data sequence [14,17,18,19,20,21]. When the difference exceeds the preset threshold, an event is activated. The other is statistically based and includes both the generalized likelihood ratio (GLR) [22] and the sequential probability ratio test (SPRT). In terms of SPRT, the bilateral CUSUM algorithm is applied as a sliding window to implement the transient event detection [23,24]. Compared with the bilateral CUSUM algorithm, event detection algorithms based on the standard deviation multiple (SDM) can achieve a smaller detection error for multiple types of loads and are not sensitive to simultaneous resolution [25]. However, event detection algorithms based on SDM are not only time consuming but are also prone to falsely detect appliances with multiple starting stages and sensitive to power oscillations during stable stages. Moreover, enormous supporting appliances have been introduced as part of smart homes for their comfortableness and customized functionalities. For instance, physical and chemical/gas sensors have been deployed to collect various ambient information in residential areas [26], leading to a more complicated start-up and operation characteristic. SDM based event detection is no longer suitable for poor accuracy and long computation times. Computation efficiency-oriented research on event detection is rarely reported, but is nevertheless of vital importance to facilitate energy saving behaviors for end users through rapid human-machine interaction [27].

The electrical features of appliances are as unique as fingerprints are to individual human beings. Considering current and voltage as the most accessible features, the existing literature on NILM can be divided into two main categories, “high-frequency-based” and “low-frequency-based”. Low-frequency refers to features extracted at 1 kHz or less [28]. There are many types of features currently used in low-frequency-based NILMs including active/reactive power signature and its variation [29,30], effective current and effective voltage [31], etc. On the other hand, high-frequency based-NILMs contain harmonic decomposition [32], wavelets [33,34,35], and voltage-current trajectory (V-I trajectory) [36], etc. Many feature-combined NILM methods have also been developed. Welikala S. et al. proposed transient feature analysis of the response time and energy on power signatures to detect power demand and load operation [33]. Lin, S. et al proposed a NILM method based on V-I trajectory, instantaneous current and power, which indicates that the accuracy obtained by using a single feature is significantly less than multiple features- based ones [37]. With the popularity of smart home appliances, the functions have been widely enriched [38]. The appliances of the same functionality but of different models even exhibit poles apart characteristics, leading to a demand for a more finely divided feature sets and comprehensive feature extraction. Yu Liu, et al. proposed a high-order dynamic characteristics to successfully obtain the load decomposition with different aliasing appliances but is sensitive to predesigned classification parameters without robustness and scalability [39]. Herein, the human and computation cost for feature extraction, algorithm convergence time, and sample scales should be carefully considered before a high accuracy, real-time, and residential-friendly NILM is workable. In another words, if a large number of training samples is required for NILM, the feasibility and friendliness of the NILM-based products will be greatly reduced.

Load identification is the outcome of NILM, and it is also the most critical step. The load identification algorithm can be divided into mathematical optimization [34] and pattern recognition [24,25]. Further, it can be divided into supervised, unsupervised and semi-supervised algorithms. Load identification methods based on supervised pattern recognition have been widely used, including artificial neural networks (ANN) [40], support vector machines (SVM) [41], Adaboost [42], etc. In addition to achieving acceptable accuracy, the feasibility resulted from computation costs also deserves consideration. The residential users have strong cost sensitivity for purchasing energy-saving products. For instance, Chinese consumers on average are only willing to spend less than 10% of price premium for energy-saving appliances [43]. As the fundamental support for NILM, the algorithm is required to run fast while occupying less memory storage. The K-nearest neighbor (KNN), as a commonly used NILM method, has small computation requirement [44], but misjudgments occur when limited samples are available [45]. Gou, Ma, et al. proposed a modified KNN to address the sensitivity of the K-neighbor [46]. Gou, Qiu, et al. presented two local constrained representation-based KNN rules to design an improved classifier [47,48]. The former is a weighted representation-based KNN rule, in which the test pattern is considered as a linear aggregation of its samples by group. The latter is a weighted local mean representation-based KNN, where K-local means vectors of KNN coefficients per group are initially estimated and then utilized to represent the test pattern [44]. Based on the aforementioned two modified KNN methods, Yan F, et al. proposed a weighted KNN for residents NILM, which distributes weights evenly through the number of samples, achieving not only low computation, but also higher accuracy [45]. However, the accuracy of this method is still severely sensitive to the sample scales and availability.

The existing studies on NILM either lacks scalability to residential load monitoring in real-world cases or pays unaffordable attention to identification accuracy. As a result, it is currently challenging to identify the potential appliance with sparse sample and low computation cost. This paper proposes a sparse sample and real-time computation based NILM method which achieves high appliance identification performance with scalability and rapid computation. A wavelet decomposition based standard deviation multiple (WDSDM) method for event detection under complex startup process in smart homes scenarios is first proposed. Moreover, to distinguish similar appliances, the overshoot multiple is first introduced as an essential indicator, which further supports the load identification under ultra-sparse sample (only one sample required).

In summary, this paper reconsiders the essential features of residential loads for application purpose, and proposes a novel sparse sample and real-time computation based NILM method. The method is experimentally verified by collecting real-world appliance data. The total accuracy of the method is over 96% on average. The total time to identify 201 samples is 0.06 s. The training set for each type of appliances is reduced to only one as a support for ultra-sparse sample and real-time computation. The proposed method sheds light on the highly efficient, user friendly, scalable, and real-world implementable EMS in the expectable future.

## 2. Methodology

**Figure 1:**
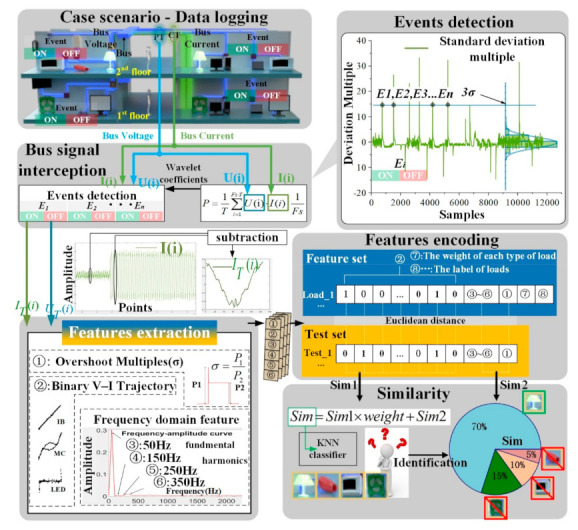
The methodology of NILM with sparse sample and real-time computation.

The overall methodology for this paper is illustrated in this section. The appliances tested and their abbreviations (also see Appendix A) are listed as follows: incandescent bulb (IB), LED lights (LED), hair dryer (HD), microwave oven (MC1 MC2), display screen (SCR1 SCR2), and electric fan (FAN). The experimental framework is illustrated in Figure 1. This paper takes the event detection, feature extraction, and load identification as three explicit steps, which are independent from each other while data are transmitted in chain. The event detection is to distinguish the switching behaviors of appliances and is non-appliance-specific (i.e., to detect any event regardless of specific type of the appliances). Once an event is detected, feature extraction comes in as the ‘fingerprint’ to extract both transient and steady state features (if applicable) for further load identification with weighted KNN. The WDSDM proposed in this paper is wavelet decomposition based only for event detection purpose, but not in terms of load identification. In this context, this paper integrates the standard deviation multiple (SDM) into wavelet decomposition for event detection as one of the contributions, which accurately and rapidly detects the events of appliances. For load identification, both V-I trajectory similarity and amplitude similarity are calculated for further integration as the comprehensive similarity. The load identification is implemented by feeding comprehensive similarity into the KNN classifier, where several confidence levels of the potential appliances are obtained as identification results.

Active power is calculated based on real-time voltage and current logging. The discrete voltage sampling value U(i) and discrete current sampling value I(i) within t seconds are collected through the data acquisition card. The method of calculating active power P generally follows formula (1). The collected data are discretized in this paper by Formula (2) to derive the power P:

(1) $$P=\frac{1}{T}\int_{-\frac{T}{2}}^{\frac{T}{2}}u(t)\cdot i(t)dt$$

(2) $$P=\frac{1}{T}\sum_{i=1}^{Fs\cdot T}U(i)\cdot I(i)\cdot \frac{1}{Fs}$$

where T is the period, and $F_{s}$ is sampling frequency.

When the event is detected, the features near the event points are extracted. Based on the active power sequence in the t time period, wavelet decomposition is performed on the active power sequence to obtain the approximation coefficient. The SDM is used to determine the change points of the approximation coefficient curve (i.e., the on time $t_{on\_i}$ and off time $t_{off\_i}$ for the i−th appliance). The sum of $t_{on\_i}$ and $t_{off\_i}$ is referred as $t_{event\_i}$.

**Figure 2:**
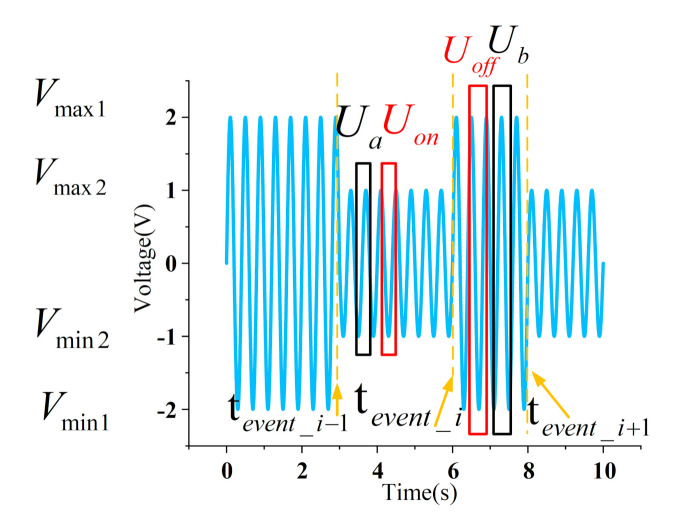
Description of voltage extraction.

As shown in Figure 2, $U_{b}$ and $U_{a}$ are extracted in period of voltage signal in the interval $\left(t_{on\_i},t_{event_{i}+1}\right)$, and the interval $\left(t_{event_{i}-1},t_{on\_i}\right)$. The FFT analysis on $U_{a}$ and $U_{b}$ is performed to obtain the phase of the fundamental component (i.e., 50 Hz). $U_{on}$ and $U_{off}$ refer to the voltage in one sampling period. The initial time of $U_{on}$ is determined after the time of $U_{a}$ at its zero phase. Similarly, the initial time of $U_{off}$ is determined before the time of $U_{b}$ at its zero phase. The current $I_{on}$, $I_{off}$ is retrieved on the same position of $U_{on}$ and $U_{off}$ from logged data. Define the abscissa as $U_{m}=(U_{on}+U_{off})/2$, and the ordinate as $I_{m}=I_{off}-I_{on}$. The V-I trajectory of the load is selected as the sub-feature ② based on $U_{m}$−$I_{m}$. The Fourier transform on $I_{m}$ is performed to extract the amplitude of fundamental, 3rd, 5th, and 7th harmonic component as sub-features ③~⑥, respectively.

**Figure 3:**
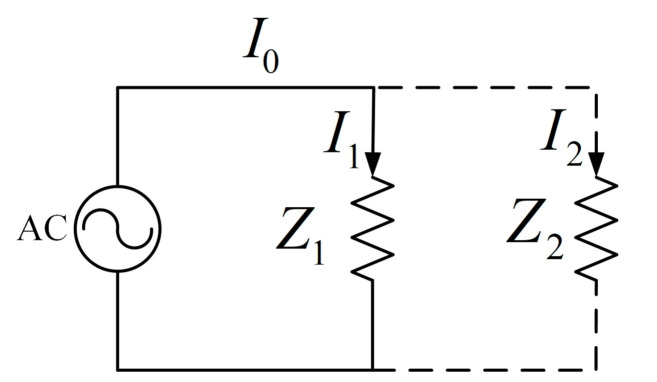
The principle of current differential manipulation.

As shown in Figure 3, since the grid responds steadily to the switching of appliances, the $U_{m}$ is regarded as an average value. Take $Z_{1}$ as the original load while $Z_{2}$ as the switching load, the difference $I_{0}-I_{1}$ indicates the stable operating current of the switching load. In this way, the operating current of the $Z_{2}$ can be derived. The current difference manipulation also avoids the harmonic interference of other appliances on the bus, and a relatively pure current of the newly switched appliance can be obtained (i.e., the harmonics injection will not affect other appliances for identification).

Both a training set and a test set are established by the feature encoding. The first 1024 (i.e., the 32 × 32 matrix of V-I trajectory is scalarized) bits of the feature is to represent the V-I trajectory. The following four bits are occupied by the sub-feature of ③~⑥ (i.e., fundamental, 3rd, 5th, and 7th harmonics). For the last three bits, the overshoot multiple, weight for each appliance, and label are inserted, respectively. In order to enrich the robustness and scalability of the dataset, data augmentation is conducted by add Gaussian noise with signal to noise (SNR) to 20 and 30, respectively.

The weighted KNN algorithm is used to identify the appliance. The comprehensive similarity is the linear combination of trajectory similarity and amplitude similarity, which are the Euclidean distance between the training sample and test sample, respectively. The identification criteria are further determined by K-nearest neighbor, which is derived from the K-largest comprehensive similarities.

## 3. Sparse Sample and Real-Time Computation Method for NILM

This section focuses on the major methods and their principles of the sparse sample and real-time computation method, including the WDSDM, the overshoot multiple, and the identification method based on weighted KNN.

### 3.1. WDSDM Based Event Detection

Wavelet transform was first proposed by Morlet and Grossman [49]. This concept was introduced to overcome the limitations of Fourier transform. Fourier transform has two shortcomings: (1) Fourier analysis cannot describe the local characteristic of signal in the time domain and (2) Fourier analysis is not good for sudden changes and non-stationary signals, since there is no time-frequency analysis. Wavelet transform can be divided into a continuous wavelet transform and a discrete wavelet transform. DWT has a structure more suitable for digital signal analysis. The continuous wavelet transform is shown as:

(3) $$CWT_{a,b}=\int_{-\infty }^{+\infty }x(t)\psi_{a,b}(t)dt$$

where $\psi_{a,b}(t)$ is called the daughter wavelet. The daughter wavelet is obtained by scaling and translating the mother wavelet (ψ(n), the wavelet function), as shown in formula (4), ψ(n) is the mother wavelet. *a* is the scaling factor and *b* is the shift factor.

(4) $$\psi_{a,b}(t)=\left|\frac{1}{\sqrt{a}}\right|\psi (\frac{t-b}{a})$$

Discrete wavelet transform is obtained by discretization of continuous wavelet transform, in which the scaling factor *a* and the shift factor *b* are discretized as (5): $a_{0}$ and $b_{0}$ are the known parameters.

(5) $$\left\{\begin{array}{l} a=a_{0}^{m}\\ b=nb_{0}a_{0}^{m} \end{array}\right.$$

Let $a_{0}=2$ and $b_{0}=1$, discrete wavelet transform is defined as:

(6) $$\begin{array}{ll} DWT_{m,n} & =a_{0}{}^{-\frac{m}{2}}\sum_{k}x[k]g[a_{0}{}^{-m}k-nb_{0}]\\ & =2^{-\frac{m}{2}}\sum_{k}x[k]g[2^{-m}k-n] \end{array}$$

**Figure 4:**
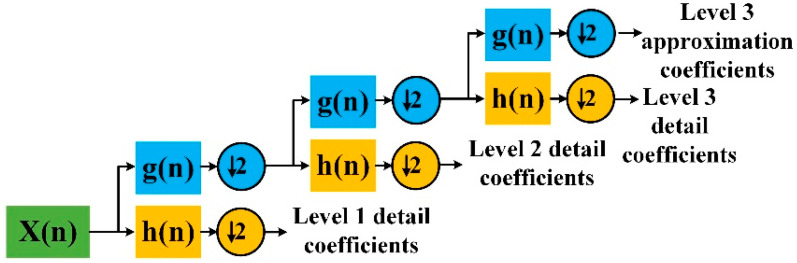
Diagram for discrete wavelet transform.

As shown in Figure 4 and formula (7), using wavelet decomposition, a signal can be decomposed into a series of signal superpositions with wavelet function and scaling function:

(7) $$x(n)=\sum_{k}c_{j_{0},k}\varphi_{j_{0},k}(n)+\sum_{j>j_{0}}\sum_{k}d_{j,k}\psi_{j,k}(n)$$

where φ(n) is the scaling function, ψ(n) is the wavelet function. The $c_{j_{0},k}$ and $d_{j_{0},k}$ are calculated by formula (8):

(8) $$\left\{\begin{array}{l} c_{j_{0},k}=\langle x(n),\varphi_{j_{0},k}(n)\rangle \\ d_{j,k}=\langle x(n),\psi_{j,k}(n)\rangle \end{array}\right.$$

One of the main applications of wavelet decomposition is signal compression and filtering. Through multi-level wavelet decomposition, the signal length can be significantly shortened, which is conducive to subsequent processing. The approximation coefficients obtained by wavelet decomposition not only retain the sudden changes caused by the input of appliances, but also suppress the fluctuation of appliances in stable operation. The use of approximation coefficients after wavelet decomposition for events detection has unique advantages.

**Figure 5:**
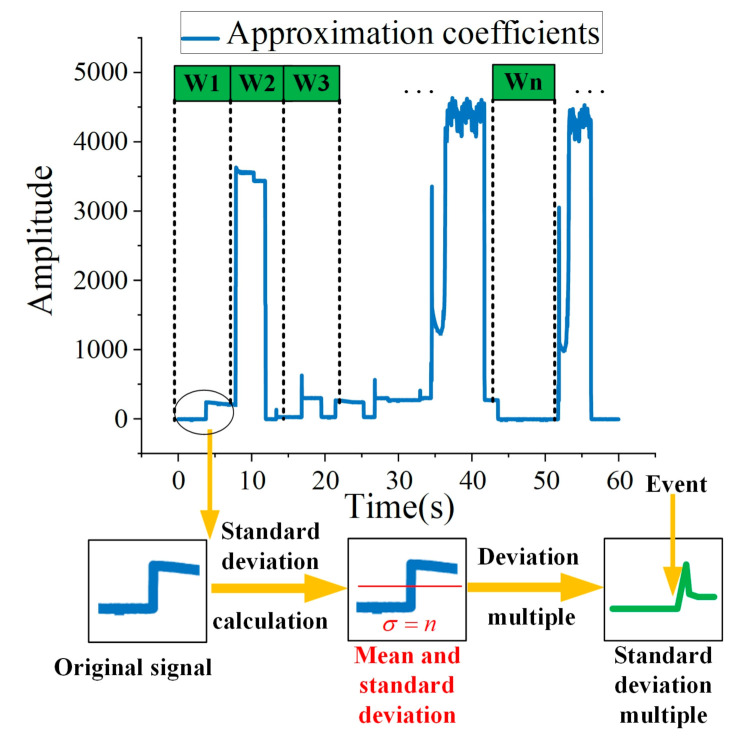
The standard deviation-based event detection.

After the active power sequence P(i) is decomposed by wavelet, App(i) is obtained. Then, the standard deviation of App(i) is analyzed. The deviation multiple of the standard deviation at the switching time of appliance is significantly higher than steady operation. Generally speaking, the threshold of event detection can reach three or more. If the standard deviation of ten consecutive points exceeds by more than three, it can be considered that there is appliance switching on behavior. Likewise, the standard deviation of ten consecutive points exceeds less than −three, it can be considered that there is appliance switching off behavior. The event detection using this method is shown in Figure 5. W1, W2, ···, Wn are the windows for mean operation. The z(i) is obtained by calculating the deviation multiple in each window for each point. When there are consecutive delay_num points greater than or less than the threshold, it is considered that there is an switching behavior of the appliance. In this method, the length m of the window and the continuous deviation length delay_num varies for different appliances.

### 3.2. Features Used in NILM

**Figure 6:**
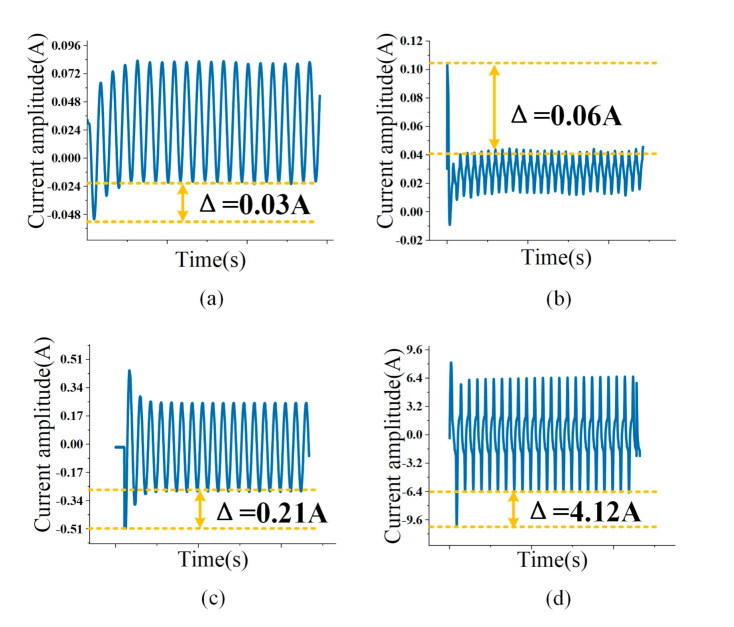
Starting current of different appliances. (**a**) 40W FAN, (**b**) 5W LED, (**c**) 45W IB, (**d**) 800W MC1.

The features used in this paper include: V-I trajectory, fundamental component amplitude, and the 3rd, 5th, and 7th harmonic component amplitudes. In addition, a new feature is also introduced as overshoot multiple to describe starting process. From the moment of starting the appliance to the stable operation, the current rises sharply and produces a spike. The starting current is appliance specific. Figure 6 shows the starting current of common appliances.

The reason for the starting current of common appliances can be explained as follows. When the incandescent lamp is started, the temperature of the resistor is low and the current will be impulsive. The current is reduced, and stability is reached after the starting process is finished. The LED lamp has a driver and a rectifier circuit, and contains capacitors. When starting the LED, the filter circuit has a short charging process, but the peak current is very large. For fans and other appliances, the starting process is longer but the peak value is low. The transient feature of active power overshoot $\sigma_{P}$ is introduced to describe different starting processes. As shown in formula (9), the physical meaning is the ratio of the maximum value of active power spike $P_{m}$ and the steady-state active power $P_{0}$ of the appliance.

(9) $$\sigma_{P}=\frac{P_{m}}{P_{0}}$$

### 3.3. Appliance Classification Based on Weighted KNN

The weighted KNN to calculate the comprehensive similarity includes the following steps: weight selection, V-I trajectory similarity and amplitude similarity, K-nearest neighbors selection, and load identification.

#### 3.3.1. The Selection of Weights

This paper modifies the KNN algorithm by introducing weights for different appliances. The sum of the weights for training samples equals 1 by appliance, and the weight is evenly assigned to each sample in the category to which it belongs. The calculation of $weight(T_{j})$ is as follows:

(10) $$weight(T_{j})=1/size(C_{T_{j}})$$

Among them, $size(C_{T_{j}})$ represents the number of training samples in the category to which $T_{j}$ belongs. $T_{1},T_{2}\;\ldots \;T_{j}$ is for different types of appliances. For instance, if IBs embraces 20 training samples in total, the weight for each IB equals 0.05 (1/20).

#### 3.3.2. V-I Trajectory Similarity and Amplitude Similarity

The V-I trajectory similarity and amplitude similarity are denoted as Sim1 and Sim2, respectively. The calculation of Sim1 and Sim2 are as Formulas (11) and (12):

(11) sim1=1/(1+dist1)

(12) sim2=1/(1+dist2)

Among them, dist1 and dist2 are the Euclidean distance of the V-I trajectory and amplitude between the two samples. Sim1 represents the similarity between samples (test set) and the training set in terms of V-I trajectory while Sim2 represents in terms of the fundamental, harmonic components, and overshoot multiples.

#### 3.3.3. The Selection of K-Nearest Neighbors

To define K-nearest neighbors, the top-K comprehensive similarity has been selected for appliance identification. The comprehensive similarity sim is determined by Formula (13):

(13) $$sim(T_{j})=sim1(T_{j})\times weight(T_{j})+sim2(T_{j})$$

#### 3.3.4. Load Identification

**Figure 7:**
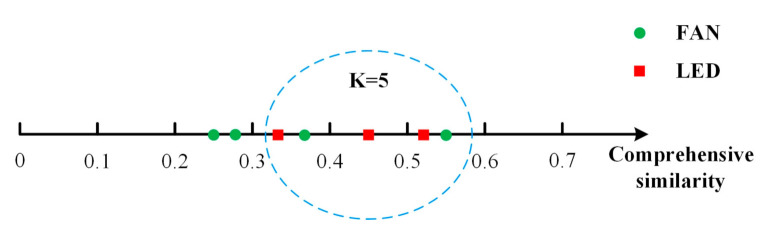
An example for load identification by weighted KNN.

The comprehensive similarity is listed in a descending order for the top-K largest ones. Further, the ratio of potential appliance is determined in this top-K nearest neighbors. In Figure 7, the K is preset as five, which means that the top five comprehensive similarity is selected for weighted KNN clustering. Among the top five comprehensive similarity, there are three potential LEDs and two potential FANs, which indicates a clustering result that the appliance is LED with a 60% (3/5) confidence.

## 4. Verification

**Figure 8:**
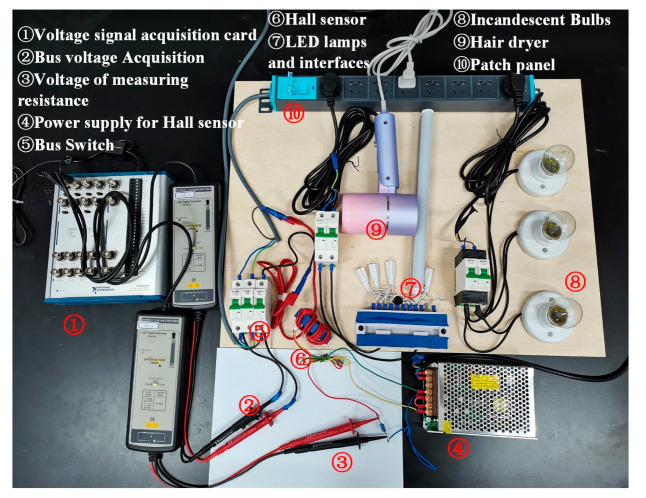
NILM experimental setup.

The NILM experimental platform is shown in Figure 8. The platform consists of a grid interface, data acquisition card, a bus splitter, processing unit, and appliances to be tested. The circuit breaker is used to control the switching of appliances. The training and testing sets are established by logging the switching data of various appliances. For load aliasing purpose, the bus splitter is used to conjunct appliances into the bus to simulate actual energy consumption scenarios.

To verify the effectiveness of proposed WDSDM for event detection, the experiment1 is designed, where 16 on-events and 16-off events are generated in a 2 min sequence by 6 types of typical household appliances. For data augmentation purpose, the samples are superposed with Gaussian noise (20 dB and 30 dB) for robustness testing under three equivalent noisy conditions. The according false alarming and detection time are recorded for comparison. In experiment two, the frequency gradient is set as 1.25 kHz, 3.125 kHz, 5 kHz and 6.25 kHz to verify the effectiveness of overshoot multiple for feature extraction. In addition, for experiment three, the frequency is located at 6.25 kHz to further verify the effectiveness of overshoot multiple for feature extraction with only one sample (ultra-sparse sample).

The sampling frequency selection is important. According to some pre-experiment, the sampling frequency is suitable at circa 6 kHz in terms of V-I trajectory distortion. When the sampling frequency is greater than 6 kHz, the V-I trajectory depicts no better graphical performance. Conversely, a frequency gradient is set as 1.25 kHz, 3.125 kHz, 5 kHz, and 6.25 kHz for robustness test for the proposed method. For the lower boundary of the sampling frequency, it is set as circa 1 kHz (1.25 kHz) since the V-I trajectory is generally categorized as a ‘high frequency’ feature by existing researches.

If the value of K selected is too small, the result of appliances identification is not trustable. If K is otherwise too large, more potential appliances will be introduced into the nearest neighbors, which may cause misjudgment. According to experience, in the application, the K value is a relatively small value and should be lower than the square root of the training sample numbers. In this experiment, K = 7.

The detailed parameters of the appliances used are shown in Table 1. The appliances can be categorized as 7 classifications by load types [50]. This paper selects the IB, HD, FAN, SCR, LED, and MC to represent a typical household energy system. These six categories of appliances also cover the power range of most residential appliances. Additionally, this paper also considers the smart home scenario, where multi-starting processes are increasingly common since more and more appliances are multi-functionated. Furthermore, the even detection is sensitive to the power fluctuation during steady state operation. The existing researches rarely considers these two points, which can be regarded as an ideal simplification to some extent. Conversely, this paper introduces the MCs and SCRs for better representativity in the household energy system scenario.

The experimental parameters are listed as below: Intel(R) Core (TM) i7-10710U CPU @ 1.10 GHz 1.61 GHz. Algorithm programming is completed based on MATLAB R2019a. The original initial sampling frequency is set to 6.25 kHz. Devices model are shown in Table 2.

## 5. Results and Discussion

In this section, the event detection and the performance of appliance identification is verified under different scenarios.

### 5.1. Performance Comparison of Event Detection Algorithms

This section compares three different event detection algorithms, including the bilateral CUSUM algorithm, the SDM based event detection and the WDSDM proposed in this paper. The indicators for comparison are time for event detection, false alarm and missed detection for both on/off switching. The time-consuming comparison is implemented by using the timing tool in MATLAB.

**Figure 9:**
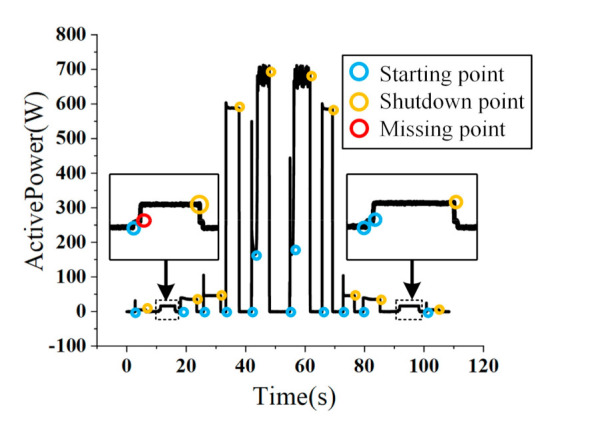
The result of event detection by WDSDM.

A sequence containing six types of appliances is generated, and three algorithms are used to detect event points. The detected points are marked on Figure 9. Table 3 shows the on/off switching sequence of each appliance. This paper uses appliances with multiple startup processes to verify the algorithm’s ability to distinguish different event points in adjacent time.

The comparison of the results of the three algorithms is shown in Table 4. The bilateral CUSUM algorithm (Algorithm 1) is of short detection time, but its false alarm rate is unacceptably high. When the appliance such as MC works stably, the active power also fluctuates greatly, so the CUSUM algorithm is easy to accumulate fluctuations in this range and cause false alarms.

The SDM (Algorithm 2) gets better accuracy, but needs to calculate the standard deviation frequently (where a large number of square operations and square root operations are required), leading to a long computation time. The event detection algorithm proposed in this paper (WDSDM, Algorithm 3) first performs wavelet decomposition on the original active power signal, obtains approximation coefficients to shorten the signal length, and also filters out some high-frequency components. The standard deviation multiple of the data except for the events point is significantly reduced. Therefore, it significantly reduces false alarm rate.

**Figure 10:**
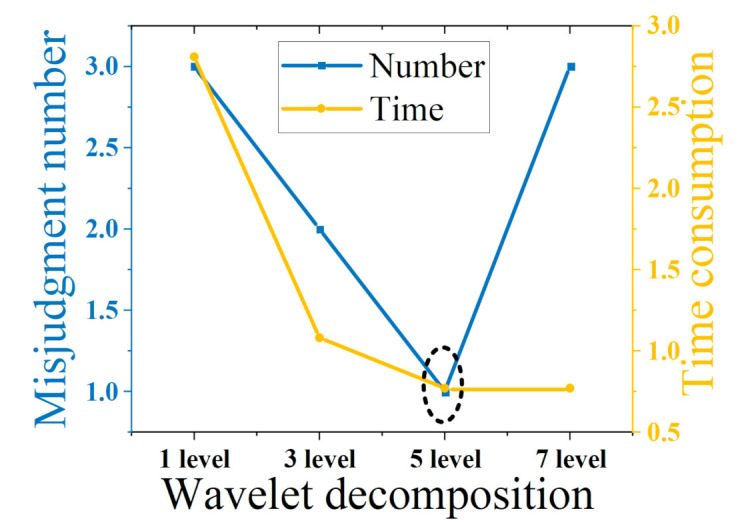
Comparison of the results for different wavelet levels.

Table 5 shows the events detection results under different wavelet decomposition levels. For the 1-level and 3-levels decomposition, as the number of layers increases, the accuracy of events detection increases, but the computation requirement decreases. When the number of layers continues to increase, the computation requirements changes little while even more mis-detections are observed. This is due to the fact that as the number of decomposition layers increases, high-frequency information is lost. Figure 10 shows that the 5-levels decomposition is optimal.

### 5.2. Research on the Improvement of Accuracy by Overshoot Multiple

To enhance the scalability and robustness of the proposed method, this paper adopts data augmentation to expand the data set. The Gaussian noise is added to the originally collected data. The original volume of the data set is n, and the data volume reaches 3n after data augmentation. The data construction and augmentation in this paper are shown in Table 6. In this experiment, K = 7.

Many appliances start up with several stages, such as MCs and SCRs, in this paper. Therefore, the starting stages of such appliances are separately regarded as different loads. The effectiveness of overshoot multiples $\sigma_{P}$ is verified by introducing two types of training sets, which represents the experiment without and with overshoot multiples as F1 and F2, respectively. F1 is the training set using V-I trajectory + harmonics, and F2 is the training set using V-I trajectory + harmonics + $\sigma_{P}$. Additionally, the experimental results are compared under different sampling frequencies.

**Figure 11:**
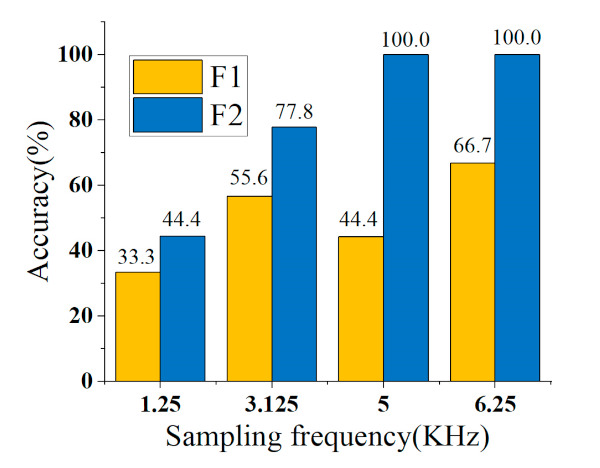
The effectiveness of overshoot multiple $\sigma_{P}$.

The identification results of the eight types of appliances are shown in Table 7. Introducing the feature of $\sigma_{P}$ improves the identification accuracy of SCR1 and LED. The higher the sampling frequency, the more obvious the improvement. If the sampling frequency is reduced, the introduction of $\sigma_{P}$ will cause certain fluctuations in the identification result, but the overall effect is positive. The identification result of the first starting point (SCR1) of the SCR is shown in Figure 11.

### 5.3. Appliance Identification under Sparse Sample

The original dataset is randomly divided into a test set and a training set. The number of training set samples is reduced to only one for each type of appliances. The test set is still half of the total data volume. The experimental results of appliance identification under sparse sample are shown in Table 8. The total time consumption is 0.06 s to identify 201 samples with accuracy of 100%. (K = 1, Sampling frequency = 6.25 kHz, train sets = 8, test sets = 201, Random seed = seed1)

**Figure 12:**
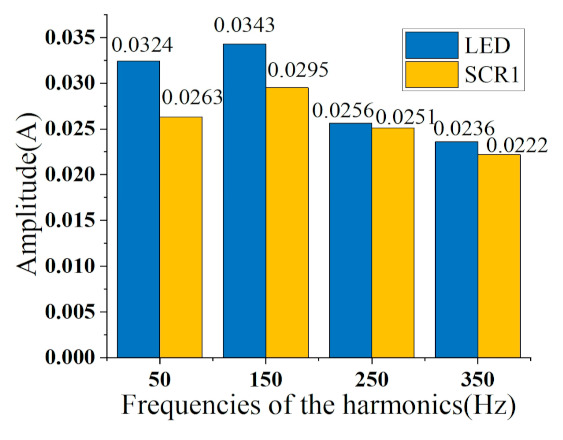
Harmonic comparison of LED and SCR1.

When the number of training sets drops to only one sample for each type of appliances, the feature of overshoot multiple proposed in this paper still has a good support for the results. The training set that does not use overshoot multiples has a more serious decline in identification results. However, it is difficult to distinguish the two in terms of V-I trajectory and harmonic amplitude, since the LED and the SCR1 share similar features. Figure 12 shows the comparison of the harmonic amplitudes of LED and SCR1. The fundamental and harmonic amplitudes of the two appliances are similar, but in terms of overshoot multiples, LED is generally above 3, while SCR1 is generally some 0.1. Therefore, the two appliances can be separated by the overshoot multiple.

### 5.4. Comparison and Discussion

There are many researches on NILM, and the comparison with other methods is shown in Table 9, where basic steps concerning event detection, feature extraction, and load identification are abbreviated as ED, FE and LI, respectively.

Event detection based on wavelet decomposition are mainly divided into three types: (1) directly, using the coefficients of wavelet decomposition as the object of event detection [55,56]; (2) using an energy distribution pattern of wavelet decomposition [57,58]; and (3) using the entropy of wavelet coefficients at different resolution levels [59]. Compared with the previous algorithm of event detection based on wavelet decomposition, the method in this paper can be classified as type (1). The approximation coefficients of wavelet decomposition are used for event detection. This method omits the calculation steps of energy and entropy, and no additional threshold calculation steps required. Therefore, the calculation time is reduced.

There are many kinds of load identification algorithms and features used for load identification. Based on these methods, most works are highly accuracy-oriented. However, there is still not much attention to calculation time and sample scale. The relatively short calculation time can reach within 1 s, and the relatively small sample scale is below 100. Compared with the previous literature, the NILM in this paper is of a real-time and ultra-sparse sample characteristic. The literature [45] also uses the V-I trajectory and harmonic amplitude. By comparison, in the case of low sample scale, the introduction of overshoot multiples in this paper can achieve higher accuracy, especially for appliances with similar V-I trajectory and harmonic compositions.

## 6. Conclusions

At present, there are many accuracy-oriented NILM researches, with rare consideration of the sample scale and computation requirement. Based on a novel WDSDM, overshoot multiples, and a modified weighted KNN, this paper implements NILM with sparse sample and real-time computation. The main contributions of this paper are as follows:

(1) This paper builds an experimental platform and uses the real-world data of residential appliances to verify the effectiveness of sparse sample and real-time computation based NILM.
(2) The WDSDM is first proposed to empower event detection of appliances with complex starting processes. The result indicates an only 1 false detection out of 16 and the time consumption is only 0.77 s.
(3) The overshoot multiple $\sigma_{P}$ is first introduced as an essential indicator for NILM. It is verified through experiment that an average identification improvement from 82.1% to 100%. Especially, the overshoot multiple facilitates over 30% identification accuracy on SCR1.
(4) Ultra-sparse sample is required for high appliance identification performance. The combination of modified weighted KNN and overshoot multiples achieves 100% appliance identification accuracy under the sampling frequency of 6.25 kHz.

Through the experimental results, it can be concluded that the ultra-parse and real-time computation NILM saves 2 s per minute. Beyond this, the overall EMS for residential loads is potential to spare 1.6 h each day. The ultra-sparse sample is smaller than ANN in sample scale [37], and thereby represents a squeezed memory storage for implementable devices in smart homes. Moreover, the ultra-sparse sample scale empowers the scalability of NILM under large scale EMS scenarios, where the storage space and computation cost are currently unaffordable. Follow-up work will focus on expanding other energy consuming scenarios, enriching the types of appliances, and also studying simultaneous starting processes in NILM with sparse sample and real-time computation.

## Figures and Tables

**Table 1:** The detailed experimental appliance parameters.

Appliance	Rated Power(W)
FAN	40
HD	650
LED	5
IB	45
MC	800
SCR	16

**Table 2:** Devices model.

Device	Model
Data acquisition card	NI USB-6361
Differential voltage probe	SI-9110
Switching power supply	Shangyuan D60- ±12
Hall current sensor	LT 58-S7
Matching measuring resistance	100 Ω

**Table 3:** The events time of appliances (Apps).

Apps	Event Time(s)	Apps	Event Time(s)
LED on	2.802	LED off	6.352
SCR on	10.725, 11.305	SCR off	16.189, 16.419
FAN on	18.097	FAN off	23.520
IB on	25.770	IB off	31.585
HD on	33.308	HD off	37.711
MC on	41.915, 43.140	MC off	47.954
MC on HD on IB on FAN on SCR on LED on	54.800, 55.499 65.685 72.794 79.495 91.170, 91.750 100.746	MC off HD off IB off FAN off SCR off LED off	61.660 69.191 76.576 85.186 98.257, 98.517 105.038

**Table 4:** Event detection results of three algorithms.

Algorithms	Time (s)	False (On)	Missed (On)	False (Off)	Missed (Off)
1	0.83	32	4	32	2
2	4.97	0	2	5	0
3	0.77	0	1	0	0

**Table 5:** Results based on different wavelet decomposition levels.

Levels	Time(s)	False Alarm (On)	Missed Detection (On)	False Alarm (Off)	Missed Detection (Off)
1	2.81	0	2	1	0
3	1.08	0	2	0	0
5	0.77	0	1	0	0
7	0.77	0	3	0	0

**Table 6:** Dataset settings.

Appliances	Original Amount	SNR = 30	SNR = 20	Total Amount
FAN	14	14	14	42
HD	24	24	24	72
LED	17	17	17	51
IB	25	25	25	75
MC1	18	18	18	54
MC2	18	18	18	54
SCR1	9	9	9	27
SCR2	9	9	9	27

**Table 7:** Appliance (Apps) identification accuracies (K = 7).

Apps	Samples	5 kHz Accuracy (%)		3.125 kHz Accuracy (%)		1.25 kHz Accuracy (%)	
		F1	F2	F1	F2	F1	F2
FAN	14	100	100	100	100	100	100
HD	24	100	100	100	100	100	100
LED	17	100	100	94.1	100	100	88.2
IB	25	100	100	100	77.8	100	100
MC1	18	100	100	100	100	100	100
MC2	18	100	100	100	100	100	100
SCR1	9	**44.4**	**100**	**55.6**	**77.8**	**33.3**	**44.4**
SCR2	9	100	100	100	100	100	88.9
Total	134	94.8	100	96.3	94.4	95.5	94.0

**Table 8:** Accuracies of the training set of a single sample.

Apps	Samples	Accuracy (%)	
		F1	F2
FAN	21	**57.1**	**100**
HD	34	100	100
LED	30	**16.7**	**100**
IB	34	100	100
MC1	30	100	100
MC2	24	100	100
SCR1	15	**86.7**	**100**
SCR2 Total	13 201	100 **82.1**	100 **100**

**Table 9:** Comparison of different NILM.

Ref.	Step	Method	Acc. (%)	Time (s)	Sample	Pros	Cons
[51]	ED	Cepstrum filtering	>97.31	0.35	-	Robust	Threshold sensitive
	FE	Multi-scale wavelet packet tree	-	-	-	Low sampling frequency	-
	LI	Ensemble bagging	>96.36	0.33	30	Satisfactory accuracy and complexity	Supervised method
[52]	ED	-	-	-	-	-	-
	FE	Improved DB9 algorithm	-	-	128 code-books	Filter the noise and low distortion	Only power feature
	LI	HMM	>92.76	-	100	Suppress data	Appliances sensitive
[53]	ED	DWT	>95.23	-	-	Both time and frequency analysis	False positive
	FE	DFT, energy spectrum	-	-	-	Rich features indicators	Sampling intensive
	LI	k-NN + SVM+ Decision tree	>93.09	-	684	Combined method	None-optimized
[33]	ED	DWT	-	-	-	-	-
	FE	DWT	-	-	-	Better than STFT	Sampling intensive
	LI	Feedforward NN	>95	<2	521	Iterations reduced	Noise sensitive
[54]	ED	-	-	-	-	-	-
	FE	Parseval energy	-	-	-	Field implementable	-
	LI	Decision Tree	>90	-	-	Simple but still accurate	Preliminary
[37]	ED	-	-	-	-	-	-
	FE	Energy spectrum	-	-	-	Transient performance	Complicate setups
	LI	ANN	>97	0.5	Period * 60 * 256	Better accuracy	Sampling sensitive
Ours	ED	WDSDM	>96.7	0.36	-	Multi-start appliances, faster detection, no preset threshold	-
	FE	overshoot multiple	-	-	-	Easy to extract, physical interpretation	-
	LD	Weighted KNN	>96	0.06	1	Ultra-sparse sample Real-time computation	Supervised learning

## Data Availability

Not applicable.

## Appendix A

Abbreviations in this paper are shown in Table A1.

**Table A1:** Abbreviations and full names in this article.

Abbreviation	Full Name	Abbreviation	Full Name
FAN	Electric fan	NILM	Non-intrusive load monitoring
HD	Hair dryer	EMS	Energy management system
LED	LED lights	ILM	Intrusive Load Monitoring
IB	Incandescent bulb	CUSUM	Cumulative Sum Control Chart
MC1	First start point of microwave oven	SDM	Standard deviation multiple
MC2	Second start point of microwave oven	KNN	K-Nearest Neighbor
SCR1	First start point of Display screen	SNR	SIGNAL-NOISE RATIO
SCR2	Second start point of Display screen	Apps	Appliances
